# Optimising targets for tsetse control: Taking a fly’s-eye-view to improve the colour of synthetic fabrics

**DOI:** 10.1371/journal.pntd.0007905

**Published:** 2019-12-12

**Authors:** Roger D. Santer, Glyn A. Vale, David Tsikire, Steve J. Torr

**Affiliations:** 1 Institute of Biological, Environmental, and Rural Sciences, Aberystwyth University, Aberystwyth, Ceredigion, United Kingdom; 2 South African Centre for Epidemiological Modelling and Analysis, University of Stellenbosch, Stellenbosch, South Africa; 3 Natural Resources Institute, University of Greenwich, Chatham, United Kingdom; 4 Division of Tsetse Control Services, Causeway, Harare, Zimbabwe; 5 Liverpool School of Tropical Medicine, Liverpool, United Kingdom; KARI-Trypanosomiasis Res Centre, KENYA

## Abstract

The savannah tsetse flies, *Glossina morsitans morsitans* and *G*. *pallidipes*, are important vectors of Rhodesian human African trypanosomiasis and animal African trypanosomiasis in East and southern Africa. We tested in Zimbabwe whether robust, synthetic fabrics, and innovative fly’s-eye-view approaches to optimise fabric colour, can improve insecticide-treated targets employed for tsetse control. Flies were caught by electrocution at a standard target comprising a 1m x 1m black cotton cloth panel with 1m x 0.5m black polyester net panels on each side. Catches were subdivided by species and sex. Tsetse catches were unaffected by substitution of the black cotton with a blue polyester produced for riverine tsetse targets. Exchanging the net panels for phthalogen blue cotton to simulate the target routinely used in Zimbabwe significantly reduced catches of female *G*. *m*. *morsitans* (mean catch 0.7 times that at standard), with no effect on other tsetse catches. However, significantly greater proportions of the catch were intercepted at the central panel of the Zimbabwe (means 0.47–0.79) versus standard designs (0.11–0.29). We also engineered a new violet polyester cloth using models of tsetse attraction based upon fly photoreceptor responses. With and without odour lure, catches of females of both species at the violet target were significantly greater than those at standard (means 1.5–1.6 times those at standard), and typical blue polyester targets (means 0.9–1.3 times those at standard). Similar effects were observed for males under some combinations of species and odour treatment. The proportions of catch intercepted at the central panel of the violet target (means 0.08–0.18) were intermediate between those at standard and typical blue polyester. Further, the reflectance spectrum of violet polyester was more stable under field conditions than that of black cotton. Our results demonstrate the effectiveness of photoreceptor-based models as a novel means of improving targets to control tsetse and trypanosomiases.

## Author summary

Tsetse flies transmit parasites that cause sleeping sickness in humans and nagana in cattle. Tsetse can be controlled using insecticide-treated fabric targets, which are typically blue or black and traditionally made of cotton. The efficiency of these targets might be improved by using modern synthetic fabrics that last longer and hold insecticide better, and by optimising the colour of these fabrics to be more attractive to tsetse. However, because flies see colour differently from humans, any attempts to do this must consider colour from a fly’s-eye-view. First, we tested a range of existing target fabrics against savannah tsetse in Zimbabwe. We found that a blue polyester currently produced for riverine tsetse targets was equally as effective in attracting tsetse as standard cotton targets, demonstrating that these more robust polyesters can be used for savannah tsetse control. We then employed novel models of tsetse attraction based upon fly photoreceptor responses to deliberately engineer a new violet polyester for greater predicted attractiveness to tsetse. In field tests, our new violet fabric attracted significantly more tsetse than a traditional black cotton or typical blue polyester. Our work shows that innovative fly’s-eye-view approaches can result in genuine improvements in tsetse control devices.

## Introduction

Tsetse flies (*Glossina* spp.) infest an estimated 10 million km^2^ of sub-Saharan Africa, and their bites transmit trypanosome parasites that inflict a significant disease burden on rural communities. Historic epidemics of human African trypanosomiasis (HAT) have killed millions of people, but a WHO-led eradication programme has now reduced global incidence to <3000 reported cases/year [1]. Meanwhile, animal African trypanosomiasis (AAT) constitutes one of the region’s greatest threats to livestock and crop production with losses to GDP estimated at >US$4.5 billion/year [2, 3]. Cheap and effective devices for tsetse control, such as artificial baits [4, 5], can help manage these diseases. There is now considerable scope for improving these baits through the use of robust modern synthetic materials and by physiologically inspired approaches to assess and improve the attractiveness of colours as flies see them [6–8].

Tsetse comprise three species groups, of which two are epidemiologically important. Riverine (Palpalis species group) tsetse transmit Gambian HAT (g-HAT) which comprises >95% of all reported cases of HAT. Savannah (Morsitans species group) tsetse are the main vectors of Rhodesian HAT (r-HAT), comprising the remaining <5% of cases, and of AAT. Savannah tsetse can be controlled using insecticide applied to cattle, or to blue/black cotton targets accompanied by odour lures. Such control is commonly recommended for the management of r-HAT and AAT [9, 10]. Control of the riverine tsetse that transmit g-HAT has not traditionally been recommended, but it is now acknowledged that it can make an important contribution to tackling the disease provided costs are kept low [10]. This imperative for high cost-effectiveness has spurred detailed investigation of the visually guided behaviour of tsetse, and the development of ‘Tiny Targets’ comprising smaller, lighter and more robust panels of blue polyester [6, 11, 12].

In Zimbabwe, effective target designs for the control of savannah tsetse have been determined both by detailed understanding of tsetse behaviour and by technical constraints. Targets are traditionally made from large panels of black or phthalogen blue-dyed cotton fabric, due to their high attractiveness to tsetse [4, 13]. Early target designs had rooves to protect their insecticides [4, 14], but subsequent identification of rain-resistant insecticides permitted the use of the roofless ‘swinger’ (S-type) target comprising a panel of black cotton fabric flanked on either side by panels of insecticide-coated polyester net to intercept circling flies [4, 14]. Because the net portions of these targets were prone to damage and fading, they were substituted with cloth panels (e.g. [15]): the greater propensity of savannah flies to land on the larger solid target area offset the disadvantage of the lack of a net to intercept circling flies [16]. Subsequently, flanking panels were made from phthalogen blue cotton, which provides a highly attractive hue for tsetse, but one that elicits landing much less effectively than black cotton [16]. This allowed insecticide to be restricted to the black cotton portion of the target, lowering the demand for insecticide [4, 16]. This ‘Zimbabwe’ target is now the standard type for deployment in field operations in that country.

In the last decade it has been realised that targets for riverine tsetse can be much smaller than those for savannah tsetse [12]. During the development of these Tiny Targets, modern blue polyesters were preferred to phthalogen blue cottons since they weigh less, so allowing easier transport and deployment [6]. These modern synthetics also last longer and hold insecticide more effectively in the field, reducing maintenance and replacement costs [6]. In addition, more robust netting materials permitted Tiny Targets to incorporate flanking nets to intercept circling flies [11]. The availability of these modern materials means that they should now be investigated for use in targets for savannah tsetse.

**Fig 1:**
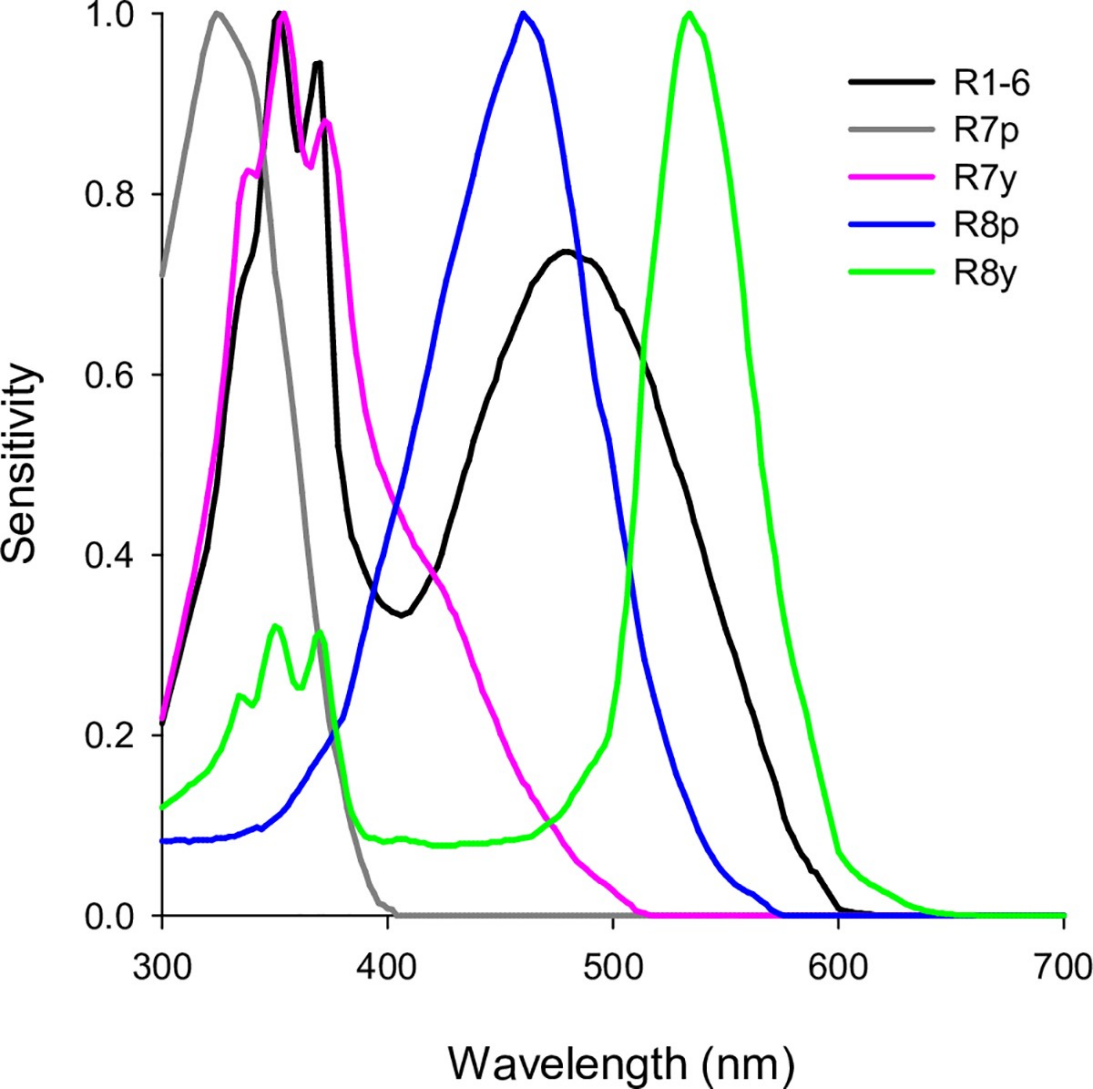
The spectral sensitivities of photoreceptors in higher flies. Flies possess five spectral types of photoreceptor across the majority of their compound eyes. The responses of these photoreceptors to light reflected from target surfaces provide the input to the visually guided behaviour of tsetse, and thus provide fly-relevant metrics of target appearance. Photoreceptor-based models statistically relate fly photoreceptor responses calculated from the reflectance spectra of coloured fabrics to the numbers of tsetse caught at traps and targets constructed from those fabrics. These models suggest that photoreceptor R7y contributes positively to tsetse attraction, while photoreceptors R7p and R8y contribute negatively [7, 8]. Such models provide a means to evaluate and refine the fly-relevant colour properties of fabrics for tsetse targets. Data are as presented in [8], based upon [18].

Colour has long been known to be an important factor in determining the attractiveness of fabric targets to tsetse [6, 13, 17]. Colour was also investigated during the development of Tiny Targets for riverine tsetse, though a polyester hue with equal or greater attractiveness to standard phthalogen blue cotton was not found [6]. In considering this issue it is essential to understand that flies do not perceive colours as humans do: higher flies possess five spectral types of photoreceptor across the majority of their compound eyes, and the responses of these photoreceptors provide the only inputs to their visually guided behaviour (Fig 1) [18–20]. Thus, a rational approach to the engineering of coloured polyesters for tsetse targets must focus on these relevant channels of sensory information, rather than human colour descriptions or raw reflectance spectra [7]. To this end, tools to calculate fly photoreceptor responses from the measured reflectance spectra of fabrics were developed [7, 8]. Using these tools, fly photoreceptor excitation values were calculated for the coloured fabrics tested in several large field studies on riverine and savannah tsetse species [6, 13, 17], and statistically related to tsetse attraction recorded in those studies [7, 8] (c.f. [20, 21]). As a result, fabric colour properties can now be described according to the sensory information actually available to a fly, and these metrics can be combined into a single predictor that scales with attraction. These photoreceptor-based models can be used to evaluate fabrics theoretically, which is important as control operations reduce fly numbers and make extensive field-testing difficult. More importantly, because it is possible to determine the reflectance spectra that would result from particular dye recipes theoretically [22], these can be evaluated and refined *in silico* [8]. In this way, the deliberate engineering of polyester fabrics for improved attractiveness to tsetse is possible.

In this study we evaluate the effectiveness of current and prospective target designs for the savannah tsetse, *Glossina morsitans morsitans* and *G*. *pallidipes*. We first compare the effectiveness of traditional cotton S-type and Zimbabwe targets with a target of the S-type design constructed using the more robust synthetic fabrics currently employed for control of riverine tsetse. We then use photoreceptor-based models of tsetse attraction to develop a new violet polyester with greater predicted attractiveness to tsetse than blue polyesters tested so far, and test it in field experiments versus the standard black cotton and a typical blue polyester alternative using the S-type target configuration. This provides the first experimental test of photoreceptor-based model predictions, and the first attempt to use them to design more effective fabrics for tsetse control.

## Methods

### Target fabrics

Standard black cotton and phthalogen blue cotton fabrics were sourced from a stock used to construct tsetse control devices in Zimbabwe (Rekomitjie Research Station, Zimbabwe). The blue polyester used in ZeroFly Tiny Targets by Vestergaard, which are designed for the control of riverine tsetse, was obtained from Vestergaard SA (Lausanne, Switzerland). The latter fabric was coated with insecticide and UV protectors, exactly as it would be deployed for tsetse control.

**Fig 2:**
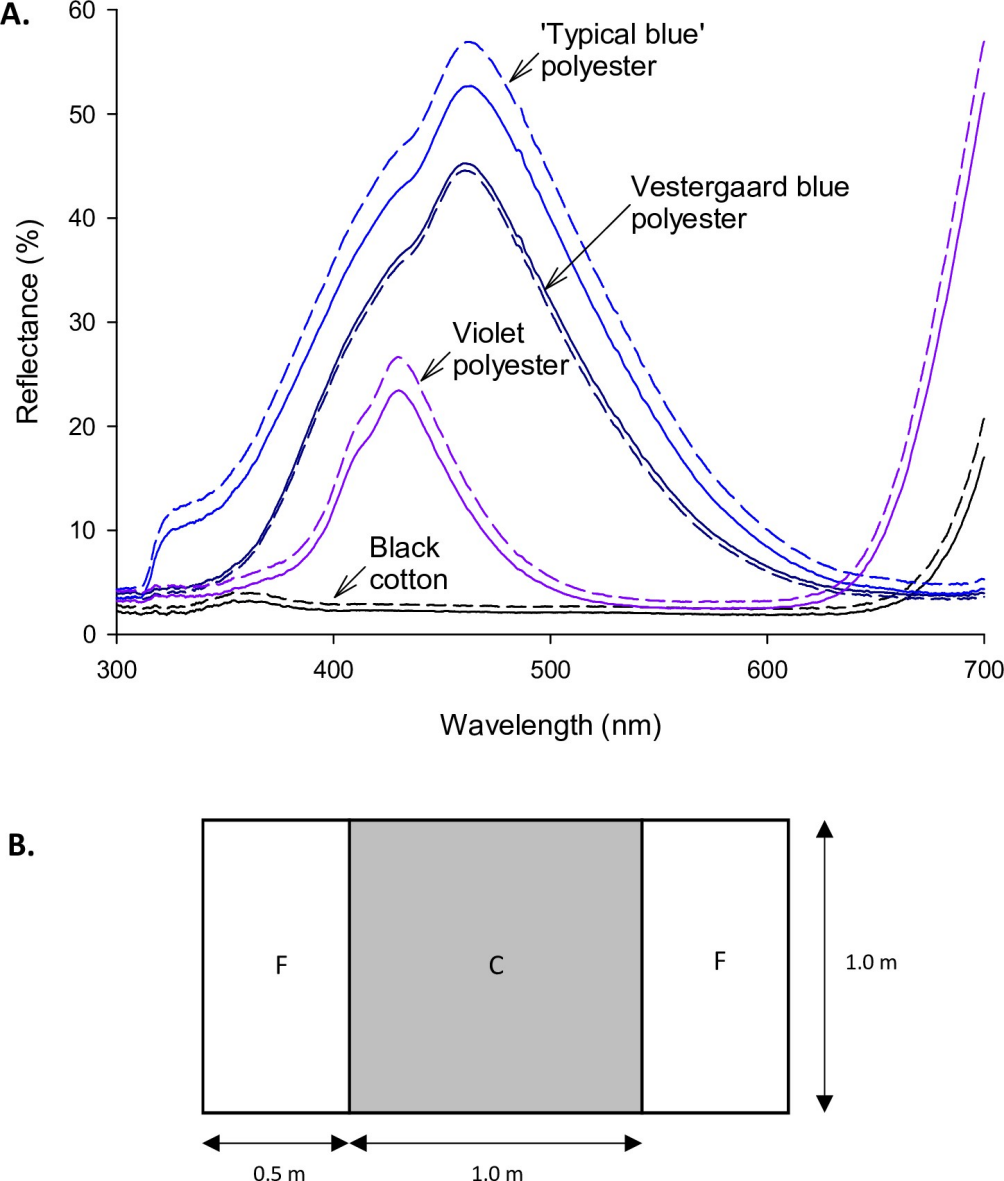
Fabric reflectance and target design. (A) Reflectance spectra for fabrics comprising the main panel of targets. Black cotton is the standard material for tsetse control devices in Zimbabwe, and ‘Vestergaard blue’ polyester is the fabric currently used in that company’s ZeroFly Tiny Targets for riverine tsetse. ‘Typical blue’ polyester and violet polyester were produced specifically for the current work by dyeing the same base polyester fabric and thus controlling for fabric properties other than colour. In particular, violet polyester was designed for increased attractiveness to tsetse using photoreceptor-based models. Solid lines are reflectance spectra with the reflectance probe oriented parallel to the bottom edge of the fabric; dashed lines are spectra recorded with the probe oriented at 90° to the bottom edge. Because spectra were similar for each side of the fabric, an average across the two sides has been plotted. (B) Fabrics were tested using the same target design which comprised a central fabric panel (‘C’) and flanking panels to each side (‘F’). The fabrics comprising these panels in each target design are listed in Table 1.

We developed two new fabrics using ‘Jupiter’ polyester microfibre with density 78 decitex and 72 filaments, and a weight of 80g/m^2^, which were produced by Toray Textiles Europe Ltd. (Mansfield, UK). Development of these fabrics is described in S1 Appendix. The first of these fabrics was dyed with CI Disperse Blue 60 at a dye bath concentration of 2.5%. This was intended to mimic the reflectance spectra of blue polyesters tested in previous experimental work which typically have a shoulder of reflectance in the UV which is thought to limit their attractiveness to tsetse [6–8, 13, 17]. For this reason we call our fabric ‘typical blue’ polyester, to distinguish it from the improved Vestergaard SA product mentioned above. We employed previously published photoreceptor-based models of tsetse attraction to develop a fabric which we expected to have improved attractiveness to tsetse versus our typical blue (S1 Appendix). This polyester was dyed using CI Disperse Violet 57 at a dye bath concentration of 7%, and for this reason we refer to it as violet polyester. We note that this fabric had significant reflectance at longer wavelengths (see Fig 2A) which aligns with the human colour description ‘purple’, but is insignificant with respect to fly vision. Because typical blue and violet polyesters used the same base fabric, their weave and surface properties were identical. This was important because the surface properties of synthetic fabrics have been proposed to be a confounding factor in studies of tsetse attraction to coloured fabrics [23].

**Table 1:** Target designs tested in the three experiments of the current work.

Experiment	Target name	Central panel	Flanking panels	Odour lure
1	Black	Black cotton	Black terylene net	AOP
	Zimbabwe	Black cotton	Phthalogen blue cotton	AOP
	‘Vestergaard’	ZeroFly blue polyester*	ZeroFly black net*	AOP
2	Black	Black cotton	Black terylene net	AOP
	Blue	Typical blue polyester	Black terylene net	AOP
	Violet	Violet polyester	Black terylene net	AOP
3	Black	Black cotton	Black terylene net	None
	Blue	Typical blue polyester	Black terylene net	None
	Violet	Violet polyester	Black terylene net	None

The reflectance spectra for central panel fabrics are shown in Fig 2A. All targets had the standard design shown in Fig 2B. AOP = acetone, octenol, 4-methylphenol and 3-n-propylphenol.

*Fabrics coated with UV protectants and impregnated with insecticide.

We quantified the reflectance of fabrics comprising the main panel of each target using an Ocean Optics USB 4000 spectrometer, PX-2 pulsed xenon light source flashing with a 30ms period, reflectance probe, and WS-1-SL standard (Ocean Optics Inc., Largo, FL, USA). The reflectance probe was angled at 45° to vertical to capture diffuse reflectance, and the distance between probe tip and fabric sample was 6mm. We used 120ms integration time, a boxcar width of 5, and averaged 25 scans to smooth data. Fabric samples were folded to give eight layers, and three replicate reflectance spectra were measured at random points on each side of the fabric and averaged for each fabric. We found that the angle of azimuth of the reflectance probe affected the overall brightness of the spectra recorded. We, therefore, recorded spectra with the reflectance probe oriented at both 0° and 90° azimuth, relative to the bottom edge of the fabric sample. Reflectance spectra for the fabrics tested in this study are shown in Fig 2A.

### Target design and field experimentation

We field-tested the effectiveness of the selected fabrics using a standard target configuration comprising a 1.0 x 1.0 m central fabric panel, flanked on each side by a 1.0 x 0.5 m net or fabric panel (Fig 2B). We tested two sets of three targets in separate experiments (Table 1). In experiment 1, targets comprised the standard black cotton S-type and Zimbabwe targets, plus a modified target similar in design to the S-type but constructed using the modern synthetic fabrics produced for ZeroFly Tiny Targets for riverine tsetse (Vestergaard SA, Lausanne, Switzerland). These targets were presented with odour lure (acetone, octenol, 4-methylphenol and 3-n-propylphenol; c.f. [24]), as is standard for savannah tsetse (Table 1). In experiments 2 and 3, we tested our newly developed polyester microfibre fabrics and the standard black cotton S-type target used in experiment 1. Experiment 2 presented these targets with accompanying odour lure, but no odour lure was presented in experiment 3 with the expectation of exposing a more pronounced colour effect (Table 1).

Field trials were conducted at Rekomitjie Research Station, Zimbabwe. At this location, rain falls mainly between December and March, making these months unsuitable for fieldwork. The coolest months are typically June and July in the early dry season, when the average daily maximum is ~ 28°C. The hottest months are typically October and November in the late dry season, when the average daily maximum is ~ 36°C. Experiment 1 was conducted from April to October 2016; experiment 2 was conducted in two blocks spanning September to November 2017, and May to June 2018; and experiment 3 was conducted from June to August 2018. In each experiment, targets were sited as recommended by [25]. Targets were aligned with their axis running north-south, across the axis of the prevailing wind which blew to the west. Odour dispensers were placed on the ground 30cm to the west of the target. To maximize catches, the target sites were chosen so that the odour dispensers and target were not shaded by trees when catching occurred in the late afternoon. To intercept tsetse, the fabric panels of the targets were overlaid with electrocuting wires to sample flies contacting the target [26]. These fell into a collecting tray below the target, and their position in the collecting tray was used to determine the particular panel at which the flies made contact, on the assumption that electrocuted flies fell straight down. Each experiment comprised Latin squares of 3 targets x 3 days x 3 sites, and catches were recorded in the last 3 hours before sunset on each catching day. The three sampling days within each Latin square were consecutive wherever possible. Occasionally, technical issues caused data collection to be abandoned, and in these cases work resumed as soon as possible. Experiments 1 and 3 were conducted at a single location, whilst data collection for experiment 2 occurred at two locations, making six sites in total. On each sampling day, catches were separated according to sex and species. Although we did not specifically target muscoid flies, we quantified catches of these divided into biting and non-biting species. We conducted 29 Latin squares for experiment 1, 17 such squares for experiment 2, and seven for experiment 3.

### Data analysis

For each experiment, we analysed the catches of tsetse and muscoid flies at each target on each sampling day using generalized linear mixed models (GLMMs) implemented using the glmmTMB package [27], for R [28]. To assess the overall attractiveness of targets we analysed the total numbers of flies caught at each target and its flanking nets combined on each sampling day. Catches were assumed to follow a negative binomial distribution (NB2), and a log link function was chosen. Trap design was designated a fixed factor, and experimental day and site were each assigned a random intercept. For experiment 2, location was also assigned a random intercept. To assess the tendency of flies to alight directly on the target, rather than attempting to circle around it, we analysed the proportion of the total catch that was intercepted at the central panel. We call this the centre proportion. Binary logistic GLMMs were applied using numbers of flies on the central panel as successes, and numbers on the flanking panels as failures. Again, trap design was designated a fixed factor, and experimental day and site were each assigned a random intercept. For experiment 2, location was also assigned a random intercept. For both types of GLMM, post-hoc LSD tests were applied using the emmeans package [29].

## Results

### Comparison of existing target technologies

We first compared tsetse catches at targets currently employed for savannah tsetse control in Zimbabwe, and at a modified design using modern synthetic fabrics developed for the Tiny Targets that are deployed for the more cost-effective control of riverine tsetse in West and Central Africa (see Fig 2; Table 1). These targets were presented with odour lure.

**Fig 3:**
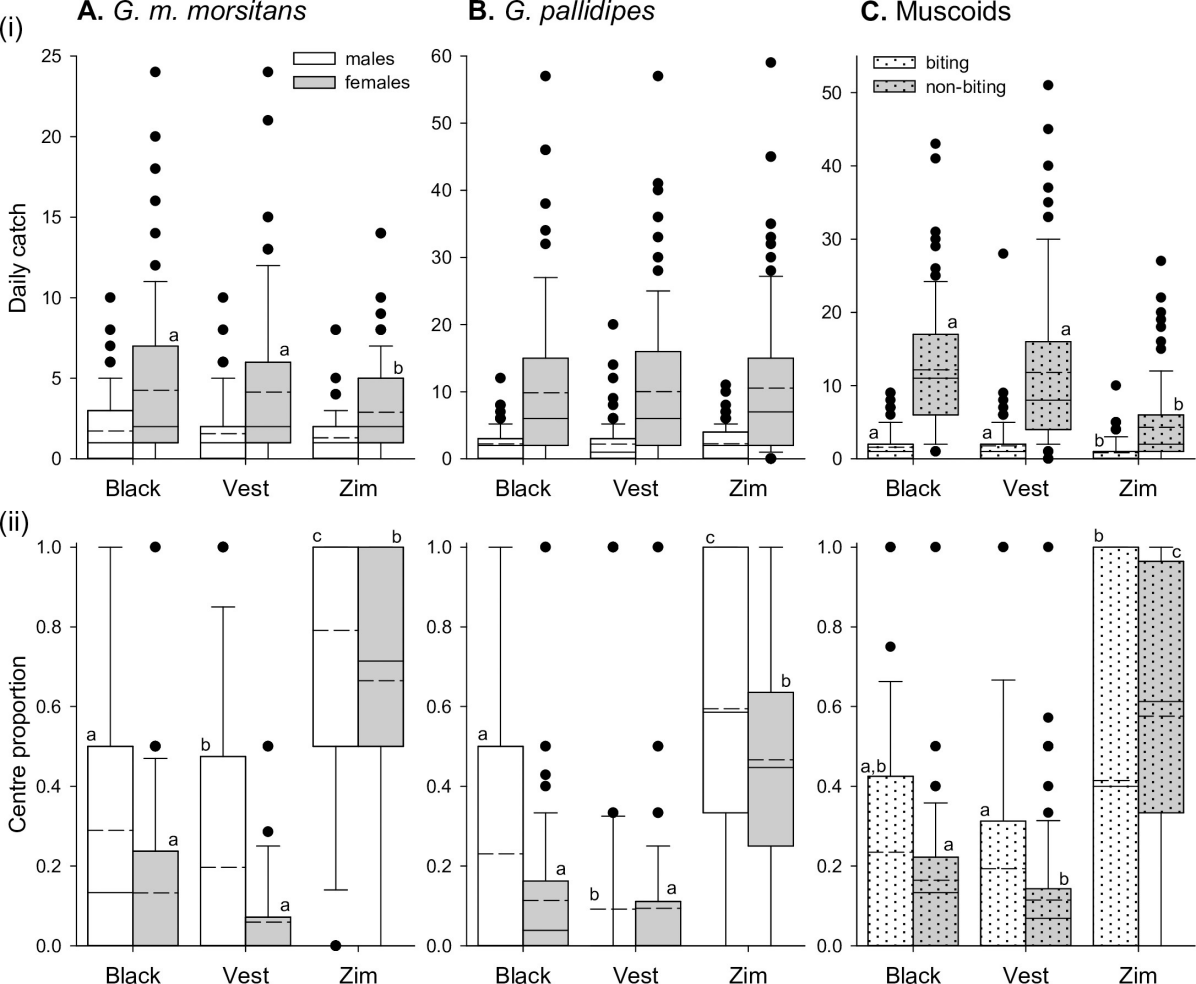
Catches of biting flies at three target designs based on existing technologies. Data are plotted for male and female *G*. *m*. *morsitans* (A), and *G*. *pallidipes* (B), and for biting and non-biting muscoid species (C). (i) Upper panels show daily catches recorded on each sampling day. (ii) Lower panels show the proportion of the catch that was intercepted at the centre panel on each sampling day. Target designs are described in Table 1 (Vest = ‘Vestergaard’; Zim = Zimbabwe), and data are from a total of 29 Latin squares encompassing 87 sampling days. Boxes enclose 25^th^ and 75^th^ percentiles, with median indicated by solid line, and mean indicated by dashed line. Whiskers indicate 10^th^ and 90^th^ percentiles, and outliers are plotted. Different letters indicate significant differences (p<0.05) as indicated by post-hoc LSD tests. Omnibus tests are reported in text.

Considering catches across the entire target area (including flanking panels of net or cloth), the three target designs performed broadly similarly for tsetse species (Fig 3A and 3B, upper panels; Table 2). There were no significant differences in daily catches of male *G*. *m*. *morsitans*, or *G*. *pallidipes* of either sex (Negative binomial-Log GLMMs; Male *G*. *m*. *morsitans*:- Wald X^2^_2_ = 3.063, p = 0.216; Male *G*. *pallidipes*:- Wald X^2^_2_ = 0.589, p = 0.745; Female *G*. *pallidipes*:- Wald X^2^_2_ = 0.842, p = 0.656; Fig 3A and 3B, upper panels). For female *G*. *m*. *morsitans*, daily catches did differ across the three target designs (Wald X^2^_2_ = 13.598, p = 0.001). For these flies, catches at the black cotton and Vestergaard blue polyester S-type targets did not differ significantly from each other, but significantly exceeded those at the Zimbabwe-type target (Fig 3A, upper panel). Mean catches of female *G*. *m*. *morsitans* at the black cotton and Vestergaard blue polyester targets were 1.5 and 1.4 times that at the Zimbabwe design.

**Table 2:** Total numbers of flies caught over 87 sampling days of experiment 1, the number caught on the centre target panel, and that number expressed as a proportion of the total.

		*G*. *m*. *morsitans*		*G*. *pallidipes*		Muscoids	
Target		Male	Female	Male	Female	Biting	Non-biting
**Black**	**Total**	**150**	**370**	**194**	**857**	**139**	**1057**
	Centre	42	43	42	64	43	169
	*Proportion*	*0*.*28*	*0*.*12*	*0*.*22*	*0*.*07*	*0*.*31*	*0*.*16*
**‘Vestergaard’**	**Total**	**136**	**360**	**192**	**872**	**153**	**1026**
	Centre	24	28	16	66	32	96
	*Proportion*	*0*.*18*	*0*.*08*	*0*.*08*	*0*.*08*	*0*.*21*	*0*.*09*
**Zimbabwe**	**Total**	**114**	**252**	**195**	**916**	**73**	**376**
	Centre	87	171	103	398	29	223
	*Proportion*	*0*.*76*	*0*.*68*	*0*.*53*	*0*.*43*	*0*.*40*	*0*.*59*

The rationale for the development of the Zimbabwe target design was the greater tendency of tsetse to alight on the central black panel against the blue flanking panels, allowing insecticide to be restricted to that central panel. We thus compared the proportion of total catches that were intercepted at the central panel (the ‘centre proportion’; Fig 3A and 3B, lower panels). Centre proportions varied significantly across the three target designs for males and females of *G*. *m*. *morsitans* and *G*. *pallidipes* (Binary-Logistic GLMMs; Males *G*. *m*. *morsitans*:- Wald X^2^_2_ = 77.220, p<0.001; Female *G*. *m*. *morsitans*:- Wald X^2^_2_ = 232.160, p<0.001; Male *G*. *pallidipes*:- Wald X^2^_2_ = 77.329, p<0.001; Female *G*. *pallidipes*:- Wald X^2^_2_ = 373.730, p<0.001). In each case, the centre proportion of the Zimbabwe target exceeded that at the other designs. For males of both species, the centre proportion at the black cotton S-type also exceeded that at the Vestergaard blue polyester S-type (Fig 3A and 3B, lower panels).

We also examined these trends for muscoid flies and identified significant variation in daily catches across the three target designs for both biting and non-biting species (Negative binomial-Log GLMMs; Biting:- Wald X^2^_2_ = 19.998, p<0.001; Non-biting:- Wald X^2^_2_ = 159.470, p<0.001; Fig 3C, upper panel). In both cases, catches at the black and Vestergaard polyester S-types did not differ significantly from each other, but significantly exceeded those at the Zimbabwe-type target (Fig 3C, upper panel; Table 2). Mean daily catches at the black and Vestergaard polyester S-type targets were *ca*. 2.7–2.8 times that at the Zimbabwe target for non-biting muscoids, and *ca*. 1.9–2.1 times that at the Zimbabwe target for biting muscoids. Centre proportions also varied significantly across the three target designs for biting and non-biting muscoids (Binary-Logistic GLMMs; biting:- Wald X^2^_2_ = 9.036, p = 0.011; non-biting:- Wald X^2^_2_ = 335.760, p<0.001; Fig 3C, lower panel). As for tsetse, centre proportions for both types of muscoid were significantly greater at the Zimbabwe-type target than the Vestergaard blue S-type. For non-biting muscoids only, centre proportions at the black cotton S-type were significantly greater than at the Vestergaard blue S-type, but significantly less than at the Zimbabwe-type target (Fig 3C, lower panel).

### Field testing of new fabrics with odour lures

**Fig 4:**
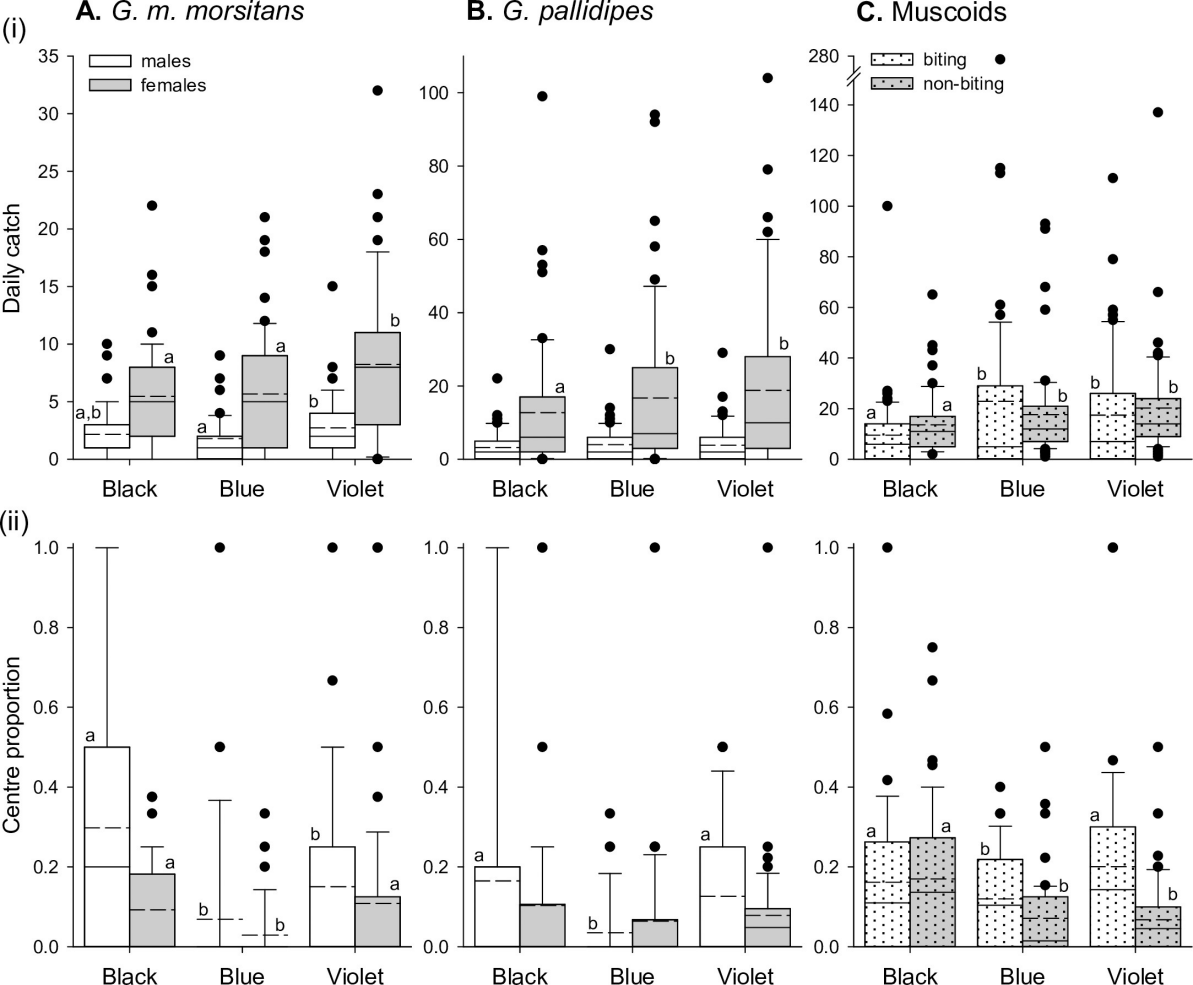
Catches of biting flies at new polyester and traditional cotton targets with odour lures. Data are plotted for male and female *G*. *m*. *morsitans* (A), and *G*. *pallidipes* (B), and for biting and non-biting muscoid species (C). (i) Upper panels show daily catches recorded on each sampling day. (ii) Lower panels show the proportion of the catch that was intercepted at the centre panel on each sampling day. Target designs are described in Table 1, and data are from a total of 17 Latin squares encompassing 51 sampling days. Figure conventions are as described for Fig 3.

Previously published photoreceptor-based models suggested that violet polyester would be more attractive to tsetse than our typical blue, and possibly also black cotton (S1 Appendix). Over all 51 sampling days of the experiment, the total numbers of *G*. *m*. *morsitans* caught at the violet polyester target were *ca*. 1.3–1.5 times those at the black cotton or blue polyester targets (Table 3). The daily catches of males and females differed significantly across the three target colours (Negative binomial-Log GLMMs; Males:- Wald X^2^_2_ = 6.704, p = 0.035; Females:- Wald X^2^_2_ = 20.907, p<0.001; Fig 4A, upper panel). For males, daily catches were significantly greater at the violet polyester than at the typical blue polyester but not at the black cotton target. Here, the mean catch of males at the violet target was 1.5 times that at typical blue (Fig 4A, upper panel). For females, daily catches were significantly greater at the violet polyester than at the typical blue polyester and black cotton targets. Again, the mean catch of females at the violet target was *ca*. 1.5 times that at the other targets (Fig 4A, upper panel). For *G*. *pallidipes*, the total numbers of flies caught at the violet target over all 51 sampling days of the experiment were *ca*. 1.0–1.5 times those at the other targets (Table 3). The daily catches of females differed significantly across the three target colours, while the daily catches of males did not (Negative binomial-Log GLMMs; Males:- Wald X^2^_2_ = 4.383, p = 0.112; Females:- Wald X^2^_2_ = 28.598, p<0.001). Catches of females at the typical blue and violet polyester targets did not differ significantly from each other, but significantly exceeded those at the black cotton target (Fig 4B, upper panel). At the violet target, the mean daily catch of female *G*. *pallidipes* was *ca*. 1.5 times that at the standard black cotton S-type target.

**Table 3:** Total numbers of flies caught over 51 sampling days of experiment 2, the number caught on the centre target panel, and that number expressed as a proportion of the total.

		*G*. *m*. *morsitans*		*G*. *pallidipes*		Muscoids	
Target		Male	Female	Male	Female	Biting	Non-biting
**Black**	**Total**	**111**	**279**	**164**	**650**	**487**	**694**
	Centre	27	34	17	45	102	117
	*Proportion*	*0*.*24*	*0*.*12*	*0*.*10*	*0*.*07*	*0*.*21*	*0*.*17*
**Blue**	**Total**	**92**	**289**	**203**	**854**	**1170**	**900**
	Centre	6	8	8	43	154	63
	*Proportion*	*0*.*07*	*0*.*03*	*0*.*04*	*0*.*05*	*0*.*13*	*0*.*07*
**Violet**	**Total**	**139**	**420**	**196**	**959**	**890**	**1034**
	Centre	17	40	26	62	220	71
	*Proportion*	*0*.*12*	*0*.*10*	*0*.*13*	*0*.*06*	*0*.*25*	*0*.*07*

Centre proportions also varied significantly across the three target colours for male and female *G*. *m*. *morsitans* (Binary-Logistic GLMMs; Males:- Wald X^2^_2_ = 12.695, p = 0.002; Females:- Wald X^2^_2_ = 13.075, p = 0.001), and for male but not female *G*. *pallidipes* (Binary-Logistic GLMMs; Males:- Wald X^2^_2_ = 10.012, p = 0.007; Females:- Wald X^2^_2_ = 2.568, p = 0.277). For male *G*. *m*. *morsitans*, the centre proportion at the black cotton target significantly exceeded that at the typical blue and violet polyester targets (Table 3; Fig 4A, lower panel). However, for female *G*. *m*. *morsitans* and male *G*. *pallidipes*, the centre proportions did not differ significantly between black cotton and violet polyester targets, but the proportions at these targets exceeded those at the typical blue polyester target (Table 3; Fig 4A and 4B, lower panels).

Although the targets under test in this experiment were not specifically intended to affect catches of muscoid flies, we identified significant variation in daily catches across the three target colours for both biting and non-biting muscoids (Negative binomial-Log GLMMs; Biting:- Wald X^2^_2_ = 18.290, p<0.001; Non-biting:- Wald X^2^_2_ = 15.572, p<0.001). For both species groups, daily catches at the violet polyester target did not differ from those at the typical blue polyester target, but the catches at these targets significantly exceeded those at the black cotton target (Fig 4C, upper panel). The mean daily catches of biting and non-biting muscoids at the violet target were *ca*. 1.8 and 1.5 times respectively those at the black cotton target. Centre proportions also varied significantly across the three target colours for biting and non-biting muscoids (Binary-Logistic GLMMs; Biting:- Wald X^2^_2_ = 48.086, p<0.001; Non-biting:- Wald X^2^_2_ = 53.984, p<0.001). Centre proportions for biting muscoids were equivalent between the black cotton and violet polyester targets, but significantly exceeded those at the typical blue polyester target (Table 3; Fig 4C, lower panel). For non-biting muscoids, centre proportions were greater at the black cotton target than either polyester target (Table 3; Fig 4C, lower panel).

### Field testing of new fabrics without odour lures

**Fig 5:**
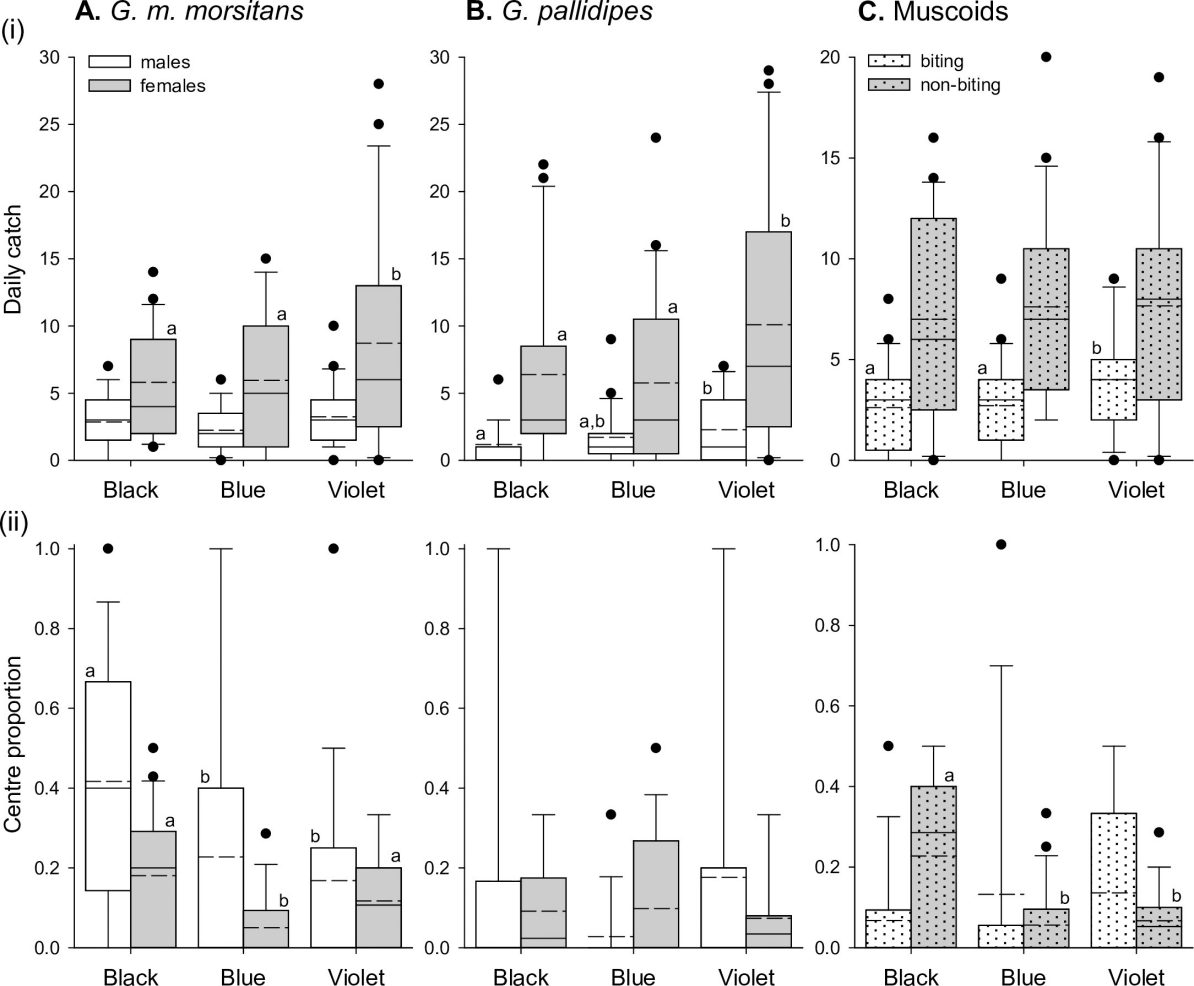
Catches of biting flies at new polyester and traditional cotton targets without odour lures. Data are plotted for male and female *G*. *m*. *morsitans* (A), and *G*. *pallidipes* (B), and for biting and non-biting muscoid species (C). (i) Upper panels show daily catches recorded on each sampling day. (ii) Lower panels show the proportion of the catch that was intercepted at the centre panel on each sampling day. Target designs are described in Table 1, and data are from a total of 7 Latin squares encompassing 21 sampling days. Figure conventions are as described for Fig 3.

We next used the targets from experiment 2 without odour lures on the basis that colour effects might be enhanced (e.g. see [30]). In this shorter experiment, total catches of male and female *G*. *m*. *morsitans* at the violet target over all 21 sampling days were *ca*. 1.1–1.5 times those at the black cotton and blue polyester targets (Table 4). Daily catches of female but not male *G*. *m*. *morsitans* differed significantly across the three target colours (Negative binomial-Log GLMMs; Males:- Wald X^2^_2_ = 2.700, p = 0.259; Females:- Wald X^2^_2_ = 16.021, p<0.001). For females, daily catches were significantly greater at the violet polyester than typical blue polyester and black cotton targets (Fig 5A, upper panel); the mean catch at the violet target was *ca*. 1.5 times that at the other targets. For *G*. *pallidipes*, total catches at the violet target over all 21 sampling days were *ca*. 1.3–1.9 times those at the black cotton and blue polyester target (Table 4). There were significant differences in daily catches of both sexes across the three targets (Negative binomial-Log GLMMs; Males:- Wald X^2^_2_ = 6.473, p = 0.039; Females:- Wald X^2^_2_ = 13.516, p = 0.001) (Fig 5B, upper panel). For males, catches at the violet target significantly exceeded those at the black cotton target, with a mean catch at the violet target of 1.9 times that at black cotton; for females, catches at the violet target significantly exceeded those at the black cotton and blue polyester target, with mean catches *ca*. 1.6 and 1.8 times respectively those at the other targets.

**Table 4:** Total numbers of flies caught over 21 sampling days of experiment 3, the number caught on the centre target panel, and that number expressed as a proportion of the total.

		*G*. *m*. *morsitans*		*G*. *pallidipes*		Muscoids	
Target		Male	Female	Male	Female	Biting	Non-biting
**Black**	**Total**	**60**	**122**	**25**	**134**	**55**	**147**
	Centre	27	25	4	12	5	36
	*Proportion*	*0*.*45*	*0*.*20*	*0*.*16*	*0*.*09*	*0*.*09*	*0*.*24*
**Blue**	**Total**	**47**	**125**	**36**	**121**	**57**	**160**
	Centre	11	7	2	5	7	10
	*Proportion*	*0*.*23*	*0*.*06*	*0*.*06*	*0*.*04*	*0*.*12*	*0*.*06*
**Violet**	**Total**	**68**	**183**	**48**	**212**	**84**	**161**
	Centre	12	24	5	13	10	12
	*Proportion*	*0*.*18*	*0*.*13*	*0*.*10*	*0*.*06*	*0*.*12*	*0*.*07*

Centre proportions varied significantly across the three target colours for *G*. *m*. *morsitans* (Binary-Logistic GLMMs; Males:- Wald X^2^_2_ = 11.921, p = 0.003; Females:- Wald X^2^_2_ = 11.032, p = 0.004), but not for *G*. *pallidipes* (Binary-Logistic GLMMs; Males:- Wald X^2^_2_ = 1.681, p = 0.431; Females:- Wald X^2^_2_ = 2.436, p = 0.296) (Table 4; Fig 5A and 5B, lower panels). For male *G*. *m*. *morsitans*, the centre proportion at the black target was significantly greater than that at the blue and violet polyester targets, whilst for females, centre proportions were equivalent at the black and violet targets and significantly exceeded those at the blue polyester target.

We identified significant variation in daily catches across the three target colours for biting but not non-biting muscoids (Negative binomial-Log GLMMs; Biting:- Wald X^2^_2_ = 6.781, p = 0.034; Non-biting:- Wald X^2^_2_ = 0.702, p = 0.704). For biting muscoids, catches at the violet polyester target significantly exceeded those at the typical blue polyester and black cotton target (Fig 5C, upper panel). For biting muscoids, the mean catch at the violet target was *ca*. 1.5 times that at the other targets. Centre proportions varied significantly across the three target colours for non-biting but not biting muscoids (Binary-Logistic GLMMs; Biting:- Wald X^2^_2_ = 0.372, p = 0.830; Non-biting:- Wald X^2^_2_ = 25.751, p<0.001). Centre proportions for non-biting muscoids were significantly greater at the black cotton than at the typical blue and violet polyester targets (Table 4; Fig 5C, lower panel).

### Evaluating the robustness of newly developed fabric colour properties

**Fig 6:**
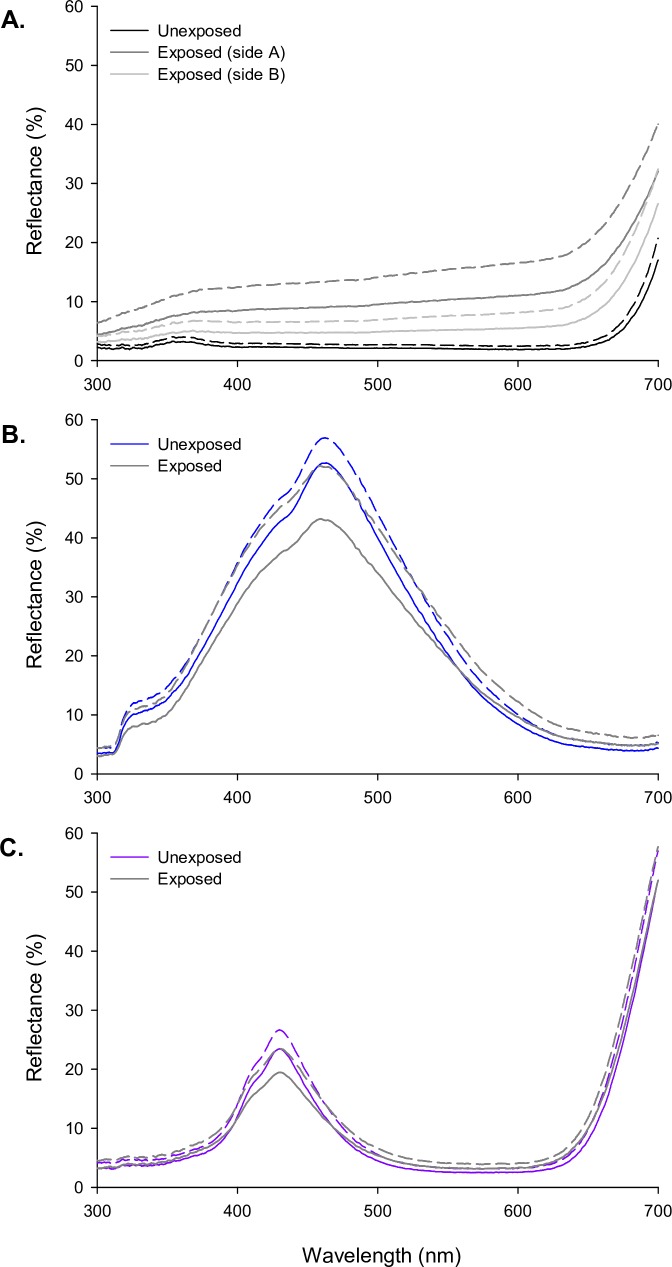
The effect of field exposure on fabric reflectance spectra. (A-C) Each panel shows the reflectance spectra recorded for unexposed (black and coloured lines), and exposed (grey lines) fabric samples. As in Fig 2, reflectance spectra were measured with two reflectance probe orientations to the weave of the fabric, and for both sides of the fabric. Spectra have been averaged within each probe orientation across the two sides for the polyester fabrics, but not for black cotton for which the exposure effect was notably greater on one side of the fabric, presumably relating to its orientation in the field.

During experiment 2, fabrics were deployed for 51 days of catching, from September 2017 until June 2018. At completion of data collection we re-measured the reflectance spectra for the fabrics tested to investigate the robustness of colour (Fig 6). Over this period, the black cotton fabric had faded considerably, and there was a notably greater extent of fading on one side of the tested fabric compared to the other (Fig 6A), probably resulting from differential shading due to target orientation in the field. The typical blue and violet polyesters were also affected by exposure, but to a much lesser extent than the black cotton, and principally by dirtiness rather than fading. There was a slight decrease in peak reflectance for both fabrics, and a slight increase elsewhere in the spectrum (Fig 6B and 6C). Evaluated via our photoreceptor-based models, the attractiveness of the black fabric was predicted to be most severely affected by exposure, reducing to levels similar to the typical blue polyester on the most faded side (see S1 Appendix).

## Discussion

In this study we investigated potential improvements in target design for the control of savannah tsetse that might be transferred from studies of riverine tsetse. We confirmed the earlier finding that the Zimbabwe target currently deployed for control operations in that country is advantageous when insecticide application must be restricted, but found that the more robust target fabrics developed for riverine tsetse can make effective targets for savannah species. However, we also found that a new violet polyester deliberately engineered for greater attractiveness according to the predictions of photoreceptor-based models can yield significant improvements in attractiveness over black cotton and/or typical blue polyester S-type targets. The colour properties of this fabric were also relatively more robust than those of the standard black cotton under field conditions. As such, photoreceptor-based models have considerable potential for the evaluation and development of more attractive fabrics for tsetse control devices, and the dogma that blue and black are the most attractive hues for tsetse is challenged.

Our first experiment confirmed previous findings for the standard black cotton S-type target and Zimbabwe target [16]. The total catch of the Zimbabwe target was only significantly less than that of the standard S-type for female *G*. *m*. *morsitans* among tsetse, though it performed less well than the S-type for both biting and non-biting muscoids. However, as anticipated, the proportion of that catch intercepted at the central panel which would bear insecticide during normal deployment was significantly greater than for the S-type designs for all tsetse and for non-biting muscoids.

The new synthetic fabrics developed for riverine tsetse can also make effective targets for savannah species when deployed in the S-type configuration, since we found total catches at such targets to be statistically indistinguishable from those of the standard black S-type for all fly species examined. With such targets, the lesser centre proportion is not a disadvantage as the entire target would bear insecticide. The performance of such targets, coupled with the increased robustness and insecticide-holding potential of new fabric and net materials may make these targets viable alternatives to the Zimbabwe target, especially since the fabrics can be insecticide-coated during manufacture. The further elucidation of these matters requires more detailed study of the robustness of new materials under field conditions, and the way they affect the economics of target deployment and maintenance.

In addition to testing existing target technologies, we also tested a new violet polyester developed using photoreceptor-based models of tsetse attraction, and a typical blue polyester with a shoulder of UV reflectance similar to the blue polyesters that Lindh et al. [6] found to be less attractive to riverine tsetse than phthalogen blue cotton. Catches of female tsetse in the presence and absence of odour lures were significantly greater at the violet fabric than at the black cotton standard and, with the exception of female *G*. *pallidipes* in our second experiment, at the typical blue polyester target. The pattern for males was less consistent, but the violet fabric significantly outperformed typical blue polyester for male *G*. *m*. *morsitans* in the presence of odour lure, and it outperformed the black cotton standard for male *G*. *pallidipes* in the absence of odour. Since we have standardised the weave of the polyester fabrics tested here, the observed differences in attractiveness between typical blue and violet polyesters must result from differences in their spectral reflectance, free of the structural effects proposed to explain previous trends in attraction to coloured synthetic fabrics [23]. The blue polyester used in ZeroFly Tiny Targets by Vestergaard lacks a shoulder of UV reflectance and is an improvement on the blue polyesters tested by Lindh et al. [6]. This fabric was expected to be more attractive than our typical blue polyester, but not as attractive as violet polyester (see S1 Appendix). A direct comparison between violet polyester and the Vestergaard blue polyester was beyond the resources of this study, but catches at the violet target significantly exceeded those at the black cotton S-type for several fly groups whilst during our first experiment, catches at the Vestergaard blue polyester target were statistically indistinguishable from those at the black cotton S-type for all groups examined. Therefore, the new violet polyester appears to be an effective fabric, even in comparison to the commercially produced fabric developed for riverine tsetse.

Photoreceptor-based models predicted that the black cotton target would be more effective than the typical blue polyester one (see S1 Appendix). However, catches at these targets were statistically indistinguishable for most combinations of tsetse species, sex, and odour condition, and catches of female *G*. *pallidipes* at the typical blue polyester target significantly exceeded those at the black cotton S-type in the presence of odour lure. We offer two possible explanations for this discrepancy. Firstly, the black cotton faded considerably during field trials, and the predicted attractiveness of faded black cotton was similar to typical blue polyester and much less than unexposed black cotton (S1 Appendix). Secondly, it is likely that photoreceptor-based models require refinement for more exact prediction of attraction in specific species and contexts, and dedicated experimental work will be needed to achieve this.

In previous work, 90% of the invertebrates caught at electrified targets comprised tsetse, muscoids, and tabanids, with a minimal by-catch of other groups [31]. We caught few tabanids, but analysed patterns for muscoids for comparison to tsetse. For biting muscoids, catches at the violet polyester target exceeded those at the black cotton and typical blue polyester targets in the absence of odour lure, which was consistent with predictions and observations for tsetse. However, all targets performed similarly for non-biting muscoids in the absence of odour, and in the presence of odour catches at typical blue and violet polyesters were similar, and significantly greater than those at black cotton. Surface texture is an important factor affecting the attraction of *Stomoxys* and other biting flies [32, 33], and in our study typical blue and violet polyesters were similar in that respect but different to black cotton, providing a possible explanation for the latter pattern. In addition, it is conceivable that the colour properties influencing attraction of tsetse and muscoids differ subtly. Previous work using the Nzi trap configuration found similarities in the types of blue fabric that are attractive to tsetse and *Stomoxys* [32]. However, for sticky traps–which also differ from fabrics in the important respect of surface texture–blue and black traps performed poorly against *Stomoxys* versus white, grey, green, and yellow ones [34]. Therefore, further detailed study of the mechanistic basis of attraction in biting muscoids, and development of bespoke photoreceptor-based models, might help improve coloured targets for them. Nevertheless, we emphasise that among the designs tested, our violet fabric was also effective against biting muscoids, providing a possible additional benefit to its use. The extent to which species of no epidemiological importance are represented in the by-catches of non-biting muscoids will be deserving of attention in future work.

Aside from attractiveness, a second advantage of our violet fabric is its relative effectiveness in eliciting direct landing responses, which we quantified via centre proportions. Black surfaces are known to elicit direct landing responses of tsetse more effectively than blue surfaces, and this property is exploited by the Zimbabwe target design [16]. However, centre proportions at the black cotton target significantly exceeded those at the violet polyester target only for male *G*. *m*. *morsitans* in the presence and absence of odour. Meanwhile centre proportions at the violet target exceeded those at the typical blue one for female *G*. *m*. *morsitans* and male *G*. *pallidipes* in the presence of odour, and for female *G*. *m*. *morsitans* in the absence of odour. Similar trends were broadly evident for biting muscoids, though for non-biting species the black target elicited landing responses more effectively than the violet and typical blue polyesters. It has previously been suggested that direct landing responses of tsetse reflect the same chromatic mechanism that drives attraction, alongside an additional luminance-driven mechanism influenced by UV reflectance [35]. As such, the performance of the improved fabric with respect to eliciting landing responses was not intended, but is not wholly unexpected. Although this property is not especially important with the S-type target configuration, it may indicate the advantage of improved polyesters for other trap designs where landing responses are crucial.

Our results demonstrate the utility of photoreceptor-based models in the deliberate engineering of fabrics more attractive to tsetse, and show that the colour properties of the violet fabric developed in this study are both robust and effective for targets against savannah tsetse. However, unlike Vestergaard SA’s blue polyester, the violet fabric is not a commercial product, so that before it can be used in the manufacture of targets its ability to hold coatings of insecticide and UV protectants must be evaluated. Further, we emphasise that this fabric was developed using a single dye primarily to prove concepts. We expect that further improvements will be possible by applying the same procedures to investigate a wider range of dyes, and the effects of dye mixtures; Moreover, we believe that elaborated photoreceptor-based models will help to improve predictions.

## Supporting information

S1 Appendix Fabric development and evaluation. A description of the methods employed to develop the new polyester fabrics tested in this study, including their evaluation using photoreceptor-based models of tsetse attraction.
(DOCX)

S1 Dataset The complete dataset analysed in this study. (XLSX)
