# Supplementary material for: Optimising targets for tsetse control: Taking a fly’s-eye-view to improve the colour of synthetic fabrics
Source: PLoS Negl Trop Dis. 2019 Dec 12;13(12):e0007905. doi: 10.1371/journal.pntd.0007905 (PMC6907749; doi:10.1371/journal.pntd.0007905)
Supplement: S1 Appendix — A description of the methods employed to develop the new polyester fabrics tested in this study, including their evaluation using photoreceptor-based models of tsetse attraction. (DOCX) [file pntd.0007905.s001.docx]

**S1 Appendix. Fabric Development and Evaluation**

**S1.1 Overview**

Previous work presents methods and tools for calculating fly photoreceptor excitations from the reflectance spectra of fabrics [1, 2]. That work also presents statistically determined relationships between those calculated photoreceptor excitation values, and the attraction of tsetse to traps and targets in prior field studies [1, 2]. Here we applied these models to deliberately engineer new fabrics with theoretically improved attractiveness to tsetse.

**S1.2 Theoretical determination of fabric reflectance spectra**

We first obtained samples of ‘Jupiter’ polyester microfibre dyed with varying concentrations of several different dyes (supplied by Toray Textiles Europe Ltd., Mansfield, UK). We focussed on a range of blue, violet, and fluorescent dyes since their utility was suggested by earlier work [1, 2]. These dyes included Teratop Violet BL 150%, Terasil Blue BGE-01 150%, and Terasil Red 3BFF (Huntsman Textile Effects, Singapore), Dianix Blue KFBL, Dianix Turquoise XF, Dianix Dark Blue SE-3RT, Dianix Violet S-4R, and Dianix Luminous Pink 5B (DyStar, Singapore), and Ultraphor Whitener HF (BASF, Germany). We measured reflectance spectra for dyed fabric samples according to the procedure described in our main text. We next determined the relationship between dye concentration and reflectance spectrum for each dye using single-constant Kubelka-Munk theory (c.f. [2, 3]). For each spectrum, at each wavelength step, measured reflectance, *R*, was converted to *K/S* (the ratio of absorption, *K*, to scattering, *S*, coefficients), as follows:

$$\frac{K}{S}=\frac{{(1-R)}^{2}}{2R}$$

*K/S* values are assumed to relate approximately linearly to dye concentration (expressed as %, meaning g dye per 100g fabric). Lines of best fit were calculated through the *K/S* values for the different concentrations of the dye, and these were constrained to pass through the *K/S* value of the undyed fabric. The effect of varying dye concentration on *K/S* was, therefore, modelled at each wavelength step by:

$${\frac{K}{S}}_{dyed}={\frac{K}{S}}_{undyed}+bC$$

Where *b* is the slope of the relationship between dye concentration and *K/S*, and *C* is the dye concentration (%). We also modelled combinations of different dyes by assuming the *K/S* values for dye mixtures to be the sum of *K/S* values for individual dyes. Thus, combinations of different dyes were modelled and investigated by:

$${\frac{K}{S}}_{dyed}={\frac{K}{S}}_{undyed}+b_{1}C_{1}+b_{2}C_{2}+b_{3}C_{3}$$

Because the linear relationship of *K/S* and dye concentration is expected to break down when a fabric becomes saturated with dye, a new set of samples were produced so that we could compare predicted with measured reflectance spectra for recipes determined to be of interest (Toray Textiles Europe Ltd., Mansfield, UK).

Using this approach we specified a ‘typical blue’ polyester which was intended to mimic the reflectance spectra of typical blue polyesters that have been tested in previously published work with riverine tsetse [4]. These fabrics (but notably not the fabric developed and used by Vestergaard SA in the production of ZeroFly® Tiny Targets) typically possess a shoulder of reflectance in the UV which is thought to limit their attractiveness to tsetse (see figure 2A in main text) [1, 2, 4-6]. Our ‘typical blue’ polyester was dyed with CI Disperse Blue 60 at a dye bath concentration of 2.5% (Terasil Blue BGE, Huntsman Textile Effects, Singapore).

**S1.3 Development of a fabric with improved predicted attractiveness using photoreceptor-based models.**

A tool that calculates fly photoreceptor excitations from fabric reflectance spectra was made available with previous work, and the underlying procedures are fully described therein [2]. That work also presents a range of statistically significant relationships between those calculated photoreceptor excitations and catches of tsetse at traps and targets in prior field studies [1, 2]. Therefore, to evaluate a real or theoretical fabric, we calculated the fly photoreceptor excitations its measured or calculated reflectance spectrum would elicit using the published calculator [2], and using those excitations we calculated the linear predictors of tsetse catch specified in earlier work [1, 2].

To develop a putatively more attractive fabric than typical blue polyester, our procedure was to modify dye recipes to identify the dyes and their concentrations that would result in an increase in catch predictor values versus equivalents calculated from the reflectance spectrum of our typical blue. Using these procedures, we developed a violet fabric dyed using CI Disperse Violet 57 at a dye bath concentration of 7% (Teratop Violet BL, Huntsman Textile Effects, Singapore). The reflectance spectrum of this fabric is shown in figure 2A of main text.

**S1.4 Evaluating predicted attractiveness**

The photoreceptor-based models used in fabric development were originally developed using data for the sampling of a range of tsetse species, at a range of locations, and using a range of sampling devices [1, 4-6]. Unfortunately, none of these studies exactly replicated the species and target configuration employed in the current work, so we did not expect them to provide exact, quantitative predictions for the current work.

For this reason, we first applied photoreceptor-based models to predict the attractiveness of targets tested in a previous field study that took place in Zimbabwe and which used the same target configuration that we employ in present work to catch the same savannah tsetse [7]. Our intention was to ascertain how catch predictors from photoreceptor-based models would be expected to scale with catches at targets of the configuration we employ. For the purpose of illustrating these predictions in this paper, we use a simple sum of unweighted photoreceptor excitations that was shown to scale with attraction in several previous studies. This index is calculated by: +R7y –R8y –R7p [1] (see figure 1 in main text).

Green (1986) [7] presents detransformed mean daily total catches of *G. pallidipes* for three different experiments evaluating several target colours (*G. m. morsitans* catches are also presented, but were not analysed in this work because they were low). Each of Green (1986)'s experiments included a black target without odour lure; the other target colours were presented without odour lure in one experiment, and with odour lure in the other two. Reflectance spectra for these targets were obtained from [6] (on the assumption that coloured cloth and not painted cloth had been used), photoreceptor excitations for these calculated using [2], and the above opponent index of tsetse attraction calculated after [1].

We first normalised the detransformed daily mean catches from that study to the catch of a black target without odour lure that was presented in each experiment. Catches increased with the general opponent index proposed by [1], but were greater for the experiments in which odour lures were present compared to the experiment where they were not (figure S1.1A). We next normalised the same data to the catches of a black standard target without odour lure in the odour lure-free experiment, and to a black target with odour lure in the two experiments where odour lures were used. This more clearly demonstrates the scaling of normalised catches with opponent index: normalised catches increased with opponent index, but the relationship was approximately quadratic with the rate of catch increase tending to decline as opponent index increased (figure S1.1B).

We next aligned opponent index values for the fabrics tested in our experiments with these predictions (reflectance spectra for these fabrics are shown in figure 2A in main text). Violet polyester was predicted to be the most attractive fabric, with clearly increased attractiveness versus a typical blue polyester similar to those that have been tested previously [4] (figure S1.1). Opponent indices for the Vestergaard blue polyester used in ZeroFly® Tiny Targets, and for black cotton, indicated intermediate predicted attractiveness, though the non-linear relationship between opponent index and catch meant that their predicted level of performance versus the former fabrics was difficult to determine (figure S1.1). We also calculated catch predictors using the more specific regression relationships for particular studies [1, 2] (table S1.1). These predictors disagreed in their relative ranking of black cotton and Vestergaard blue polyester, and in the extent to which these fabrics exceeded the attractiveness of typical blue. However, each of these predictors was consistent in ranking the new violet as the most attractive fabric, and typical blue the least attractive (table S1.1).

**Figure S1.1: Tsetse catch prediction using photoreceptor-based models.** Data are detransformed mean daily catches of *G. pallidipes*, originally published in [7]. These come from three experiments comparing the catches of coloured targets: two in which odour lures were used, and one in which they were not. In each plot, reported tsetse catches are shown against the opponent index of [1], calculated for coloured fabric reflectance spectra in [6]. Note that if these are calculated for coloured paint reflectance spectra from the same source instead, patterns are similar. (A) Detransformed mean daily catches normalised to the catch of a black standard target delivered without odour in each experiment of [7]. Catches increase with opponent index, but are notably greater for experiments in which targets were accompanied by odour lures. (B) Detransformed mean daily catches normalised to the catch of a black target with the same odour lure condition as the other targets in each experiment of [7]. Again, catches increase with opponent index but were better fitted by a quadratic than a linear relationship (dotted lines). Open squares superimposed on the x axes represent opponent indices calculated for the fabrics tested in the current study (mean values computed across four different reflectance spectra for different sides and reflectance probe azimuth angles). Their positions suggested that the new violet polyester would be more attractive than typical blue polyester.

**Table S1.1: Alternative predictors of fabric colour attractiveness.**

| Fabric | Opponent index | *G. pallidipes* model | *G. f. fuscipes* models |
| --- | --- | --- | --- |
| Black cotton | -0.216 | 1.830 (±0.006) | 5.003 (±0.099) |
| Vestergaard blue | -0.370 | 2.229 (±0.011) | 4.958 (±0.089) |
| Typical blue | -0.573 | 1.836 (±0.028) | 4.626 (±0.044) |
| Violet | -0.089 | 2.837 (±0.033) | 5.397 (±0.113) |

‘Opponent index’ is the simple, general index of [1], and the *G. pallidipes* model is the specific 3 photoreceptor regression equation from the same work (means ± SD presented across separate models for males and females); *G. f. fuscipes* models are the mean ± SD across 12 models presented in [2]. Each model makes different assumptions about photoreceptor sensitivities and organisation, and is applied separately to males and females. The value calculated for each model is an average computed across four different reflectance spectra for different sides and reflectance probe azimuth angles.

**S1.4 Evaluating predicted attractiveness of fabrics after exposure**

Since the opponent index considered in figure S1.1 provided a reasonable prediction of our experimental data, we also applied this index to evaluate the possible effect of fabric exposure on attractiveness to tsetse. After exposure, fading was most extreme for the black cotton fabric (see figure 6 in main text). Considering the opponent index derived from calculated photoreceptor responses for the reflectance spectra shown in figure 6 of main text, the attractiveness of the black fabric was predicted to be most severely affected by exposure, reducing to levels similar to the typical blue polyester on the exposed side (figure S1.2).

**Figure S1.2: The effect of field exposure on the predicted attractiveness of fabrics.** Opponent indices were derived using photoreceptor-based models [1, 2] from the reflectance spectra presented in figure 6 of main text. The opponent index for black cotton is reduced by exposure to a greater extent than for the polyester fabrics. This indicates that an exposed black cotton is predicted to be less attractive than an unexposed one, and more similar in attractiveness to typical blue polyester.

**S1.5 Supplementary references**

1. Santer RD. A colour opponent model that explains tsetse fly attraction to visual baits and can be used to investigate more efficacious bait materials. PLoS Negl Trop Dis. 2014;8(12):e3360.

2. Santer RD. Developing photoreceptor-based models of visual attraction in riverine tsetse, for use in the engineering of more-attractive polyester fabrics for control devices. PLoS Negl Trop Dis. 2017;11(3):e0005448.

3. Berns RS. Billmeyer and Saltzman's Principles of Color Technology, 3rd edition. New York: John Wiley and Sons, inc.; 2000.

4. Lindh JM, Goswami P, Blackburn R, Arnold SEJ, Vale GA, Lehane MJ, et al. Optimizing the colour and fabric of targets for the control of the tsetse fly *Glossina fuscipes fuscipes*. PLoS Negl Trop Dis. 2012;6(5):e1661.

5. Green CH. The effect of colour on trap- and screen-oriented responses in *Glossina palpalis palpalis* (Robineau-Desvoidy) (Diptera: Glossinidae). Bull Ent Res. 1988;78:591-604.

6. Green CH, Flint S. An analysis of colour effects in the performance of the F2 trap against *Glossina pallidipes* Austen and *G. morsitans morsitans* Westwood (Diptera: Glossinidae). Bull Ent Res. 1986;76:409-18.

7. Green CH. The effects of colours and synthetic odours on the attraction of *Glossina pallidipes* and *G. morsitans morsitans* to traps and screens. Physiol Entomol. 1986;11:411-21.
